# Beam theory predicts muscle deformation and vertebral curvature during feeding in rainbow trout (*Oncorhynchus mykiss*)

**DOI:** 10.1242/jeb.245788

**Published:** 2023-10-31

**Authors:** Yordano E. Jimenez, Ariel L. Camp

**Affiliations:** ^1^ Department of Biology, Providence College, Providence, RI 02918, USA; ^2^ Department of Biology, Tufts University, Medford, MA 02155, USA; ^3^ Department of Musculoskeletal and Ageing Science, Institute of Life Course and Medical Sciences, University of Liverpool, Liverpool, L7 8TX, UK

**Keywords:** Fluoromicrometry, Epaxial, Strain gradient, XROMM, Bending

## Abstract

Muscle shortening underpins most skeletal motion and ultimately animal performance. Most animal muscle generates its greatest mechanical output over a small, homogeneous range of shortening magnitudes and speeds. However, homogeneous muscle shortening is difficult to achieve for swimming fish because the whole body deforms like a bending beam: as the vertebral column flexes laterally, longitudinal muscle strain increases along a medio-lateral gradient. Similar dorsoventral strain gradients have been identified as the vertebral column flexes dorsally during feeding in at least one body location in one fish. If fish bodies also deform like beams during dorsoventral feeding motions, this would suggest the dorsal body (epaxial) muscles must homogenize both dorsoventral and mediolateral strain gradients. We tested this hypothesis by measuring curvature of the anterior vertebral column with XROMM and muscle shortening in 14 epaxial subregions with fluoromicrometry during feeding in rainbow trout (*Oncorhynchus mykiss*). We compared measured strain with the predicted strain based on beam theory's curvature–strain relationship. Trout flexed the vertebrae dorsally and laterally during feeding strikes, yet when flexion in both planes was included, the strain predicted by beam theory was strongly and significantly correlated with measured strain (*P*<0.01, *R*^2^=0.60). Beam theory accurately predicted strain (slope=1.15, compared with ideal slope=1) across most muscle subregions, confirming that epaxial muscles experience dorsoventral and mediolateral gradients in longitudinal strain. Establishing this deformation–curvature relationship is a crucial step to understanding how these muscles overcome orthogonal strain gradients to produce powerful feeding and swimming behaviours.

## Teaser

**Summary:** Direct measurements of strain and vertebral curvature throughout the trout body confirm the epaxial muscles experience orthogonal strain gradients during feeding.

## INTRODUCTION

**Fig. 1.**
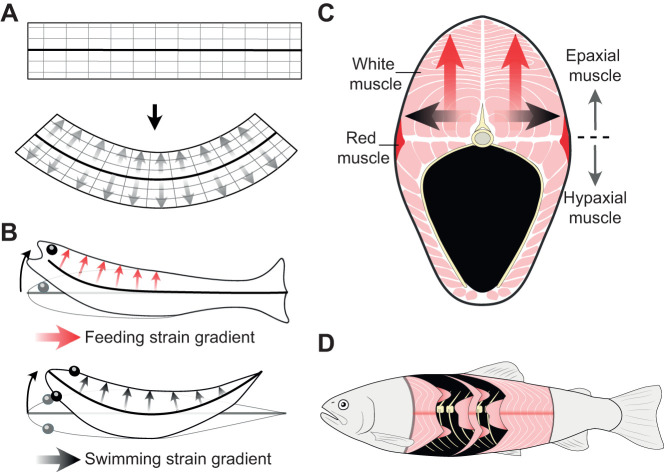
**Beam-like bending and anatomy of fish body muscles.** (A) If a simple homogeneous beam (top) is bent (bottom), longitudinal strain will increase in a linear gradient (grey arrows) with distance from the neutral axis (thick black line). (B) If fish deform like bending beams, vertebral curvature (thick black lines) will be accompanied by a dorsoventral gradient (red arrows) in longitudinal muscle strain during feeding (top) and a mediolateral gradient (black arrows) during swimming (bottom). (C) Transverse cross-section of fish body muscles (pink) and vertebral skeleton (yellow), showing orthogonal muscle strain gradients hypothesized during feeding (red arrows) and swimming (black arrows). (D) Lateral view of rainbow trout showing the epaxial and hypaxial myomeres (pink) relative to the vertebrae (yellow).

At the level of the muscle fibre, force and power are limited by intrinsic contractile force–length and force–velocity relationships. As a result, muscles can only produce high force or power over a relatively small range of fibre lengths and velocities (e.g. Altringham and Johnston, 1982; James et al., 1996, 1998; Rome et al., 1992). Therefore, muscles that operate uniformly within this small range will have a greater mechanical output. Maintaining uniform fibre strain throughout the axial muscle is particularly challenging for swimming fish (Alexander, 1969; Muller and van Leeuwen, 2006; Rome et al., 1988). Axial muscles flex the vertebral column laterally to produce powerful swimming behaviours such as sprinting and the escape response (Muller and van Leeuwen, 2006; Rome et al., 1988). As fish bend their bodies from side to side, the axial muscle tissue deforms like a simple beam (e.g. Coughlin et al., 1996; Goldbogen et al., 2005; Katz et al., 1999; van Leeuwen et al., 1990). This deformation forms a mediolateral strain gradient whereby longitudinal muscle strain at any given location is a product of both its distance from, and the curvature of, the neutral axis: the vertebral column (Fig. 1A,B). Beam-like deformation has been observed in the red (aerobic, slow-twitch) and white (anaerobic, fast-twitch) axial muscle of many species (reviewed in Long et al., 2002).

If the white axial muscle fibres that bend the body were oriented longitudinally (a ‘null’ morphology), the resulting gradient of muscle fibre strain and velocity would have a crippling effect on swimming performance. Only a small portion of the muscle fibres could operate at the optimal length and velocity for power production, limiting the overall muscle power output. Nevertheless, fish muscles are equipped with a complex gearing system that enables homogeneous fibre strain within a heterogeneously deforming muscle tissue. The white axial muscle fibres of fish are not oriented longitudinally, and instead run obliquely to the vertebral column in nearly helical trajectories (Alexander, 1969; Gemballa and Vogel, 2002; Greene and Greene, 1914). This complex fibre architecture and its deformation, including shearing of muscle fibres, is hypothesized to create a gearing system that equalizes strain of the axial muscle fibres (Alexander, 1969; Azizi and Brainerd, 2007; van Leeuwen et al., 2008). These mechanisms increase the medial fibre strain and decrease lateral fibre strain, producing uniform fibre strain while the whole muscle undergoes beam-like deformation.

In addition to fast swimming behaviours, many fishes also use the same white epaxial (dorsal half of the body) muscles to generate powerful dorsal flexion to lift the head and expand the mouth during feeding (Carroll and Wainwright, 2006; Lauder, 1985; Tchernavin, 1948; Westneat, 2006). Interestingly, feeding may also impose the same problematic gradients in longitudinal muscle strain and velocity (Jimenez et al., 2021). Similar to swimming, during feeding, axial muscles can dorsally flex large regions of the vertebral column (>30%) to produce cranial elevation (Camp, 2021). Fish may be using beam-like bending during suction feeding, wherein vertebral dorsiflexion causes the epaxials to experience a dorsoventral gradient of longitudinal muscle strain (Fig. 1B). Bluegill sunfish (*Lepomis macrochirus*) were recently shown to experience dorsoventral strain gradients during feeding and mediolateral gradients during swimming in the body region with the largest cross-sectional area (Fig. 1C) (Jimenez et al., 2021). While these strain gradients are observed over large regions spanning many epaxial myomeres, fish can independently activate different regions within each myomere (e.g. Thys, 1997; Jimenez and Brainerd, 2020, 2021). For example, largemouth bass activate only the dorsally located epaxial ‘arms’ for low performance feeding and the ventrally located epaxial ‘cones’ for low performance swimming (see Fig. 1D; Jimenez and Brainerd, 2020). However, in high performance swimming and feeding, all myomeric regions are activated along all or two-thirds of the body length, respectively (Jimenez and Brainerd, 2020). These large portions of the epaxial and hypaxial muscles (80% by mass) generate most of the power for suction feeding (Camp and Brainerd, 2022). Variable strain rates within these muscles could limit feeding power. Thus, epaxial muscles would require a specific gearing system to avoid variable fibre strain and strain rate during feeding. Given these biomechanical implications, further study into the relationship between epaxial muscle deformation and vertebral curvature during feeding is warranted.

Axial muscle deformation in feeding likely falls into one of three categories: a simple linear gradient, a complex non-linear gradient, or no gradient at all. Beam theory describes a simple linear gradient that may or may not include muscle shearing, a deformation where muscle fibres slide past each other and alter the relationship between muscle strain and vertebral curvature. Although shearing has not been empirically shown in fish axial muscles, shearing is hypothesized to occur in swimming (Goldbogen et al., 2005; van Leeuwen et al., 2008), and is likewise possible in feeding. To the extent that shear is present or absent, different models of beam theory should be favoured (Euler–Bernoulli versus Tomoshenko). The second possibility is that the complex muscular actions observed in feeding give rise to complex, non-linear gradients of muscle strain. Epaxial and hypaxial muscles are physically connected yet actuate different skeletal elements in opposite directions. Epaxials elevate the neurocranium while hypaxials retract the pectoral girdle (reviewed in Camp and Brainerd, 2022). Additionally, unlike most axial swimming behaviours, feeding often combines substantial dorsoventral and lateral flexion (Camp, 2021; Jimenez et al., 2021), perhaps disrupting simple beam-like muscle deformation.

Simple linear gradients of muscle strain are likely in regions with smooth vertebral flexion during cranial elevation (Camp, 2021; Jimenez et al., 2018), but there may be exceptions to simple beam-like bending. Vertebral curvature (Camp, 2021) and longitudinal epaxial muscle strain (Camp and Brainerd, 2014; Camp et al., 2018; Li et al., 2022) vary craniocaudally across the anterior vertebral column. For example, epaxial muscles that extend onto the rigid neurocranium must shorten in order to rotate the craniovertebral joint. Similarly, rope-like tendons can also decouple the location of muscle shortening and vertebral curvature as in the tails of thunniform swimmers (reviewed in Long et al., 2002). Species such as seahorses and pipefish, which have rope-like tendons between the neurocranium and the epaxials, may also deviate from beam-like bending during feeding (Van Wassenbergh et al., 2011). Determining whether dorsal bending in fish follows simple beam theory would help us better understand how the epaxial muscles fulfil their dual swimming and feeding roles, and how these functional demands have shaped the evolution of their anatomy and physiology.

Advances in X-ray imaging methods allow direct measurement of vertebral curvature and muscle strain during feeding. Swimming studies have successfully used dorsal or ventral view, standard (light) video to measure lateral vertebral curvature by measuring the midline of the fish (Coughlin et al., 1996; Katz et al., 1999; Shadwick et al., 1998), but this method is not reliable for measuring dorsoventral curvature (Jimenez et al., 2021). Instead, X-ray reconstruction of moving morphology (XROMM) (Brainerd et al., 2010) can be used to visualize and measure the 3D motion of the vertebral column *in vivo* (Camp, 2021). Longitudinal muscle deformation can be measured throughout many locations with fluoromicrometry: using biplanar X-ray videos to measure muscle deformation as the change in distance between radio-opaque markers (Camp et al., 2016). Previous swimming studies have typically measured longitudinal muscle strain (Coughlin et al., 1996; Goldbogen et al., 2005; Katz et al., 1999; Wakeling and Johnston, 1999) or muscle fibre strain (Ellerby and Altringham, 2001) in only 2–3 locations simultaneously with sonomicrometry. Fluoromicrometry markers in fish have been oriented to measure longitudinal strain, not muscle fibre strain (Camp and Brainerd, 2014; Camp et al., 2018, 2020; Li et al., 2022). Axial muscle fibre strain has been measured *in vivo* with sonomicrometry (Ellerby and Altringham, 2001) or post-mortem with histology (Rome and Sosnicki, 1991), but longitudinal muscle strain is the required metric for testing the predictions of beam theory (Goldbogen et al., 2005; Long et al., 2002).

In this study, we examined the relationship between white epaxial muscle deformation and vertebral curvature during feeding in rainbow trout, *Oncorhynchus mykiss*. Trout are suitable for testing this relationship because mediolateral gradients have already been observed in their epaxials during undulatory swimming and fast-starts (Ellerby and Altringham, 2001; Goldbogen et al., 2005). Trout are also morphologically distinct and phylogenetically distant from bluegill sunfish, the only other fish in which both mediolateral and dorsoventral gradients have been observed (Jimenez et al., 2021). Furthermore, feeding trout dorsally flex at least the anterior third of the vertebral column during cranial elevation (Camp, 2021). We used XROMM to measure both lateral and dorsal curvature of the anterior vertebrae during feeding strikes. At the same time, we measured deformation simultaneously at 14 locations throughout the epaxial muscles in this vertebral region using fluoromicrometry. We compared the muscle strain measurements with the predictions of Euler–Bernoulli beam theory to test (1) whether the body deforms like a beam during dorsal flexion and (2) whether the relationship between muscle deformation and vertebral curvature is consistent across vertebral regions.

## MATERIALS AND METHODS

### Animals

Epaxial muscle deformation and vertebral kinematics were measured from three adult, female rainbow trout, *Oncorhynchus mykiss* (Walbaum 1792) (hatchery reared from Kilnsey Trout Farm) using XROMM (Brainerd et al., 2010; Gatesy et al., 2010) and fluoromicrometry (Camp et al., 2016). These analyses used existing biplanar X-ray videos and computed tomography (CT) scans from a prior XROMM study (Camp, 2021), so the data collection methods are only described here briefly. The body mass and standard length of the trout were 776 g and 345 mm (Trout 1), 770 g and 340 mm (Trout 2), and 998 g and 375 mm (Trout 3). All animal husbandry and procedures were approved by the University of Liverpool Animal Welfare and Ethics Review Board and done in accordance with UK Home Office licences.

### Marker implantation

Each trout was anaesthetized with tricaine methanesulfonate (MS-222) buffered with sodium bicarbonate, and intramuscular markers were implanted into the epaxials along with an analgesic (lidocaine). Spherical tantalum markers (0.8 mm diameter; X-medics, Frederiksberg, Denmark) were injected through the bore of an 18-gauge hypodermic needle. Four series of markers (18 total) were implanted into the epaxials: left-, right- and midsagittal-superficial (left lateral, right lateral and dorsal series), and midsagittal-deep (deep series) (Fig. S1). Four bone markers (0.5 mm diameter) were implanted in the neurocranium, as described in Camp (2021). Together, the deep series and the three cranial-most dorsal series markers defined a body plane, used to approximate a fish-based frame of reference for measuring cranial elevation (Camp and Brainerd, 2014). All fish recovered fully from the anaesthetic within 30 min and resumed normal swimming and feeding behaviour within 24 h. Filming experiments were conducted at least 3 days after the surgery.

### X-ray video and anatomical data collection

**Fig. 2.**
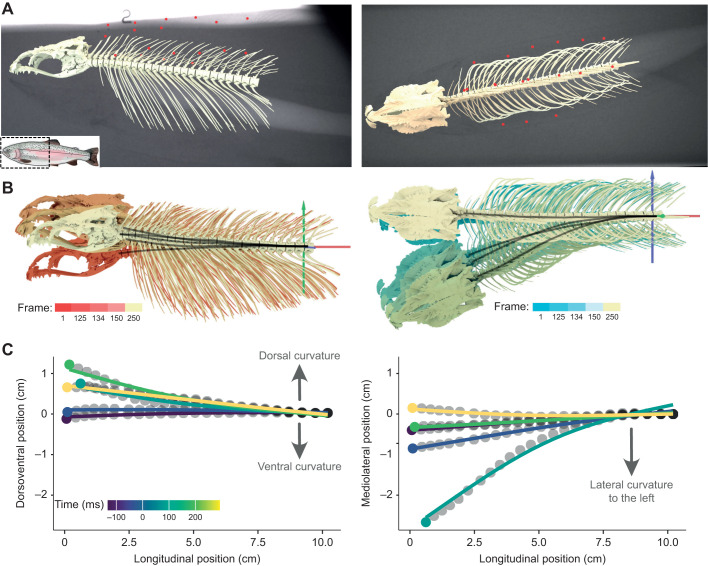
**Measurements of vertebral curvature.** (A) Frames from an XROMM animation, showing approximately lateral (left) and dorsal (right) views of the craniovertebral skeleton and epaxial markers superimposed on the X-ray images. Inset, a rainbow trout with the dashed box outlining the region measured in this study. (B) The dorsoventral (left, red-tinted images) and mediolateral (right, blue-tinted images) position of each vertebral centrum, relative to an anatomical coordinate system (red, green and blue arrows) on the caudal-most vertebra, at five frames throughout the strike. Black lines trace the midline of the centra in each view. (C) Dorsoventral and mediolateral centrum position as a function of longitudinal position (grey dots) was fitted with a smoothed spline (solid lines) to calculate curvature. Noise in the position data was smoothed to calculate curvature, the second derivative of position. Smoothing parameters were chosen *post hoc* after visual comparison of raw and smoothed values.

Fish were filmed with biplanar X-ray video while capturing non-elusive pellets or mealworms (Fig. 2A; see Camp, 2021, for full details). Dorsal and lateral view X-ray images were generated by X-ray machines (Imaging Systems and Service, Painesville, OH, USA) and recorded at 500 frames s^−1^ on Phantom cameras (M120, Vision Research, Wayne, NJ, USA). Standard grids and a calibration object were also imaged to undistort and calibrate the X-ray videos. All raw X-ray images, videos and their essential metadata are stored on the XMAPortal (https://xmaportal.org/webportal/, permanent identifier ULIVERPOOL1) in accordance with best practices for video data management in organismal biology (Brainerd et al., 2017). Ten feeding strikes were recorded from each trout, but only strikes with at least 5 deg of cranial elevation were analysed. Strikes with <5 deg cranial elevation had very low dorsoventral vertebral curvature and strain magnitudes, with low signal-to-noise ratios near the accuracy and precision limits of our study. Thus, our sample included 8 of 10 strikes from trout 1, 8 of 10 strikes from trout 2 and 4 of 10 strikes from trout 3 for a total sample size of *n*=20 strikes. All analysed strikes were submaximal, with peak cranial elevation averaging 10 deg (Camp, 2021) compared with an average of 22 deg in a previous study of rainbow trout feeding in laboratory conditions (Konow and Sanford, 2008).

Fish were euthanized with an overdose of buffered MS-222 and post-mortem CT scans were taken at 512×512 pixel resolution and 0.172 mm slice thickness (Quantum GX microCT scanner, PerkinElmer, Waltham, MA, USA). Polygonal mesh models of the markers, neurocranium and anterior 24–27 vertebrae were reconstructed in Horos (v.3.3.6, horosproject.org) and Dragonfly (v.2020.2, Object Research Systems Inc., Montreal, QC, Canada).

### X-ray video analysis

X-ray videos were undistorted and calibrated, and all intramuscular and bony markers were tracked in the open-source software XMALab (v.1.53, available at https://bitbucket.org/xromm/xmalab/src/master/) (Knörlein et al., 2016). Marker tracking precision, calculated as the mean standard deviation of marker pairs within the neurocranium, was <0.1 mm across all strikes. 3D coordinates of intramuscular markers and rigid body transformations of the neurocranium and body plane were calculated and filtered with a low-pass Butterworth filter (60 Hz cut-off). Marker-based XROMM (Brainerd et al., 2010) of the neurocranium and body plane was combined with markerless Scientific Rotoscoping (Gatesy et al., 2010) of the vertebrae to create a single XROMM animation of each strike in Autodesk Maya (v.2020, San Rafael, CA, USA) using the XROMM_MayaTools package (https://bitbucket.org/xromm/xromm_mayatools) (Fig. 2A). The intramuscular markers were added to these animations using the ‘imp’ tool.

From the XROMM animations, cranial elevation was measured as dorsal rotation of the neurocranium relative to the body plane (Camp, 2021). A joint coordinate system (JCS) was placed at the basioccipital, consisting of two anatomical coordinate systems (ACSs) with the *x*-axis oriented rostrocaudally, the *y*-axis dorsoventrally, and the *z*-axis mediolaterally. One ACS was fixed to the neurocranium and the other ACS was fixed to the body plane. Rotations about and translations along each axis of the JCS were calculated with a *zyx* rotation order and following the right-hand rule. Peak cranial elevation was calculated as the maximum *z*-axis rotation, relative to its initial value before the start of the strike, using custom scripts in MATLAB (MathWorks, Natick, MA, USA). The time of peak cranial elevation was used as ‘time zero’ to compare timings across strikes.

### Vertebral curvature measurements

**Fig. 3.**
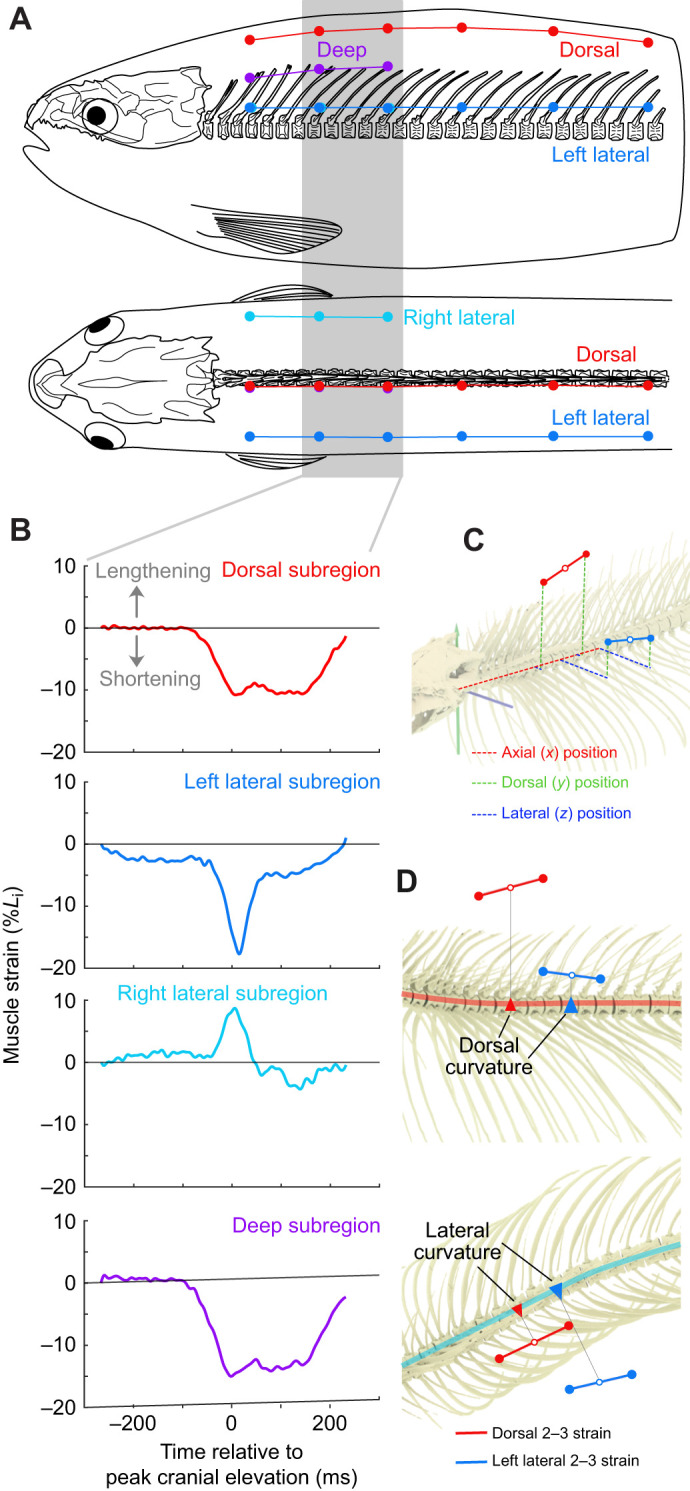
**Epaxial muscle strain measurements and predicted strain calculations.** (A) Diagram of muscle marker positions (lateral view, top; dorsal view, bottom): dorsal (red), left lateral (blue), right lateral (cyan), deep (purple) marker series. The grey bar highlights the marker pairs shown in B. See Fig. S1 for specific marker positions in each trout. (B) Muscle strain measured with fluoromicrometry from a subregion (i.e. intermarker pair) of each series during the strike shown in Fig. 2. Strain is relative to the initial length (*L*_i_) of the intermarker pair, with negative values indicating muscle shortening. (C) Calculating the position of each subregion at the initial position, demonstrated with the dorsal and lateral subregions from B. First, the axial, dorsal and lateral position (dashed lines) of each marker (filled circles) was measured relative to an anatomical coordinate system at the craniovertebral joint. Second, the positions were averaged to determine a single position for the subregion (open circles) and the *L*_i_ (solid lines). (D) Predicted muscle strain was calculated from the average position of each subregion (open circles) and the vertebral curvature at that position (triangles). Predicted dorsal (top) and lateral (bottom) strains were calculated separately and then summed.

**Fig. 4.**
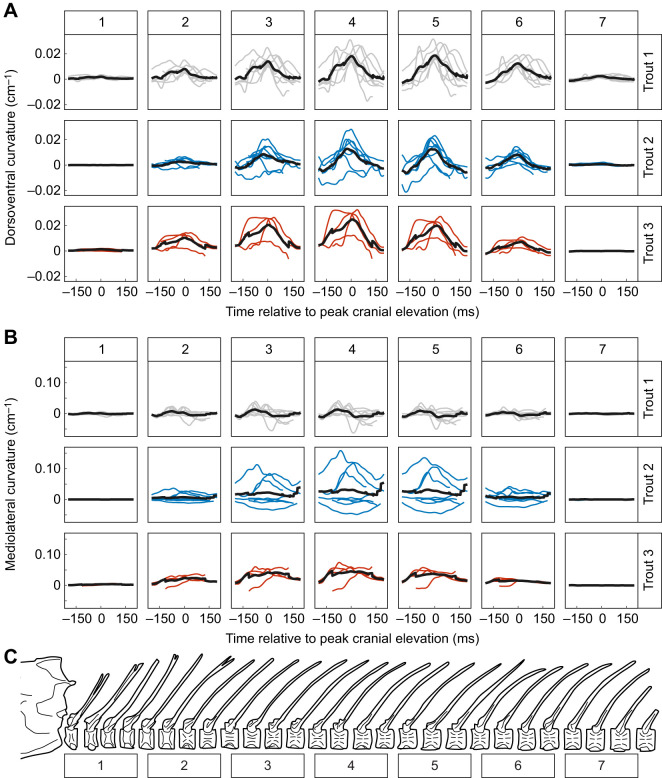
**Time-resolved curvature of the vertebral column during feeding strikes (*n*=20 strikes).** Columns show curvature from seven equidistant longitudinal positions along the vertebral column and rows show curvature data from the three individuals. For each panel, thin lines are curvature over time (relative to peak cranial elevation) for individual strikes. The thick solid line is the mean curvature at each time point across all strikes for that individual (*n*=8 strikes for trout 1 and trout 2, *n*=4 strikes for trout 3). (A) Dorsoventral and (B) mediolateral curvature calculated from the vertebral spline of the 3D centra positions over time. (C) Approximate locations of the longitudinal positions – each spanning approximately three vertebrae – relative to the vertebral column.

The motion of each vertebral centra during the strike was measured from XROMM animations. As in Camp (2021), centrum translation was measured relative to an ACS fixed to the centrum of the caudal-most animated vertebra (Fig. 2B). This provided craniocaudal (*x*-axis), dorsoventral (*y*-axis) and mediolateral (*z*-axis) positions of each centrum. At each frame, dorsoventral and mediolateral vertebral positions were each smoothed with a cubic spline of 1000 segments using the smooth.spline() function in R Studio (http://www.R-project.org/) (Fig. 2C). We used smoothed vertebral positions to calculate vertebral curvature (cm^−1^) by taking the second derivative of the spline. Lateral and dorsoventral curvature were calculated at the same craniocaudal location as each muscle subregion (see ‘Muscle strain measurements’, below, and Fig. 3) and at seven equidistant segments along the vertebral column (Fig. 4).

### Muscle strain measurements

Longitudinal strain in the epaxial muscles was measured throughout the same body region as vertebral curvature, using fluoromicrometry (Camp et al., 2016). Longitudinal strain represents muscle length changes along the craniocaudal line of action of the epaxials, not along the muscle fibres. Muscle strain was measured for each ‘muscle subregion’: adjacent marker pairs within the same marker series (Fig. 3A,B). Resting muscle length (*L*_i_) was measured once for each trout at a time prior to the strike when the vertebral column appeared least curved (Fig. S1). Muscle strain was calculated as the change in length, relative to *L*_i_, with negative values corresponding to muscle shortening (Fig. 3B).

### Calculations for predicted muscle strain

Beam theory was used to calculate the predicted strain of each muscle subregion at each frame throughout the strike. Based on the assumptions of beam theory, predicted strain can be calculated as:
(1)

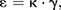
where ε is longitudinal strain, κ is curvature (cm^−1^) and γ is distance (cm) from the neutral axis of bending. Because the vertebral column is incompressible relative to the musculature, we assumed the vertebral column is the neutral axis for both dorsoventral and lateral bending. As the vertebral column often underwent biplanar flexion during feeding, we calculated the predicted strain for each plane of flexion (i.e. dorsoventral and lateral) separately to calculate the total strain. Curvature (κ) and distance (γ) were calculated for the average 3D position of each muscle pair (Fig. 3C,D). The predicted strains for each plane of flexion were added and compared against fluoromicrometry measurements of that muscle subregion using Eqn 2:
(2)

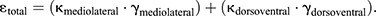


### Statistical analysis

To test the beam model of muscular deformation in feeding behaviours with simultaneous dorsoventral and lateral flexion, we corrected our data for anatomical differences in the two planes of the axial skeleton. Although the vertebral column is relatively straight within the frontal plane, the vertebral column is slightly curved in the sagittal plane at rest. Without correction, this resting curvature of the vertebral column could create the false impression that muscle at rest was either shortened or lengthened. As such, we calculated the first differences of vertebral curvature within just the sagittal plane, from which our predicted strain values were calculated.

Linear regression (model 1) was used to assess the relationship between the strain predicted by beam theory and the strain measured with fluoromicrometry, for time-resolved pooled (across all strikes and subregions) and unpooled data. The slope of this relationship indicates the accuracy of beam theory (perfect accuracy when slope=1) and the *R*^2^ value indicates precision (perfect precision when *R*^2^=1). Data were visually confirmed to follow the normality and homoscedasticity assumptions using *Q*–*Q* plots and fitted-versus-residuals plots, respectively. An equivalence test was performed using the ‘equivalence’ package to determine whether the regression results of this study are comparable to results from previous studies on beam theory, which used different experimental methods and exclusively examined fish swimming. All statistical tests were performed in R Studio.

## RESULTS

We recorded three trout performing a total of 20 submaximal feeding strikes, but with at least 5 deg of neurocranial elevation. Neurocranial elevation and dorsal flexion of the vertebral column during these feeding strikes are described in detail in Camp (2021). Despite feeding in a relatively constrained volume (10 cm wide by approximately 20 cm deep), these trout voluntarily performed a range of lateral bending behaviours during prey capture.

### Vertebral curvature

Trout flexed the anterior vertebral column laterally and dorsally (Fig. 4). Dorsoventral curvature was consistent in timing and direction: curving dorsally to reach peak dorsiflexion concomitantly with peak neurocranium elevation (time=0). In contrast, lateral curvature varied in directionality and timing: flexing left- and right-laterally either synchronously (e.g. trout 2) or asynchronously (e.g. trout 1) with neurocranial elevation and dorsiflexion (Fig. 4). The magnitude of both dorsal and lateral flexion varied across the vertebral column. The least curvature occurred in the cranial- and caudal-most regions, while the greatest curvature occurred in the middle region (approximately vertebrae 14–19). However, mediolateral curvature was usually about 2–3 times greater than dorsoventral curvature of a given region (Fig. 4).

### Muscle strain

**Fig. 5.**
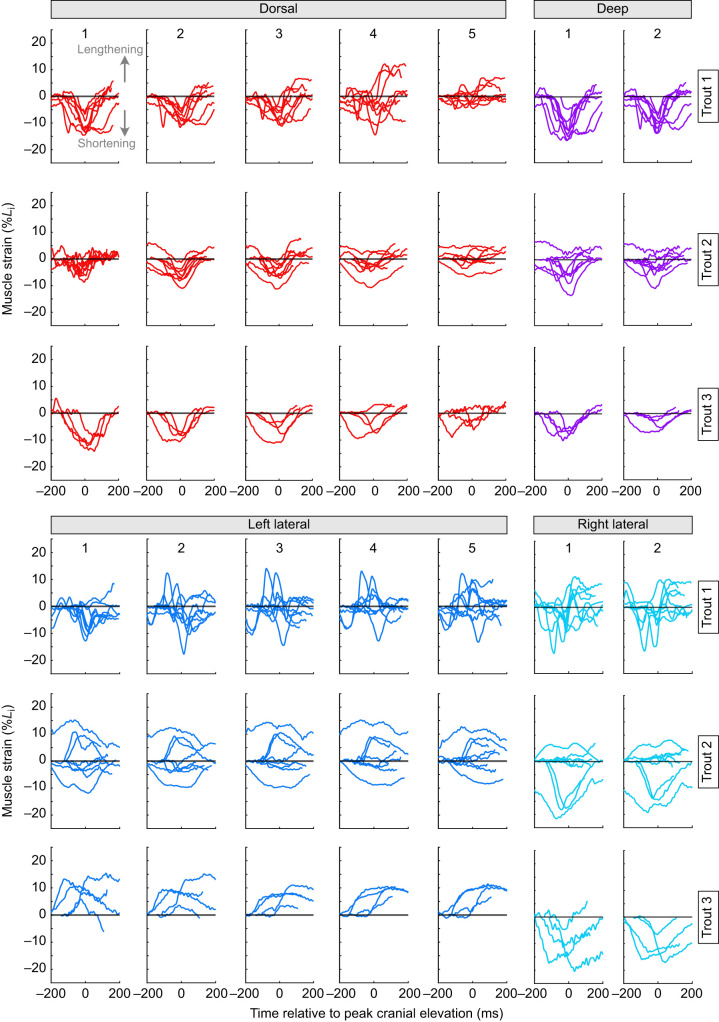
**Measured epaxial muscle strain over time during feeding strikes.** Columns show strain measured from each subregion, numbered according to longitudinal position (1=cranial-most) of the dorsal (red), deep (magenta), left lateral (blue) and right lateral (cyan) series. See Fig. 3 and Fig. S1 for the positions of each series and subregion. Rows show strain from each trout. For each graph, individual lines are the strain over time (negative values indicate shortening) for each strike from that individual, in that subregion (*n*=8 strikes for trout 1 and trout 2, *n*=4 for trout 3).

As the anterior vertebral column flexed, the white epaxial muscles changed length longitudinally (Fig. 5). The pattern of muscle strain varied between the midsagittal and the lateral muscle subregions. As with dorsoventral vertebral curvature, strain in the midsagittal muscle subregions was consistent in timing and direction (Fig. 5, top). The dorsal (midsagittal-superficial) and deep (midsagittal-deep) subregions shortened during all strikes, usually reaching peak shortening at or near the time of peak neurocranial elevation. Only the caudal-most dorsal subregion showed little or no shortening (Fig. 5, dorsal 5). As with lateral vertebral curvature, length changes in the lateral muscle subregions had variable direction and timing (Fig. 5, bottom). The left lateral and right lateral subregions (both superficial, ventral) shortened, lengthened or underwent minimal length change across different strikes. The timing of peak shortening or lengthening only sometimes coincided with peak neurocranial elevation. These strain patterns persisted even in the most caudal left subregion (Fig. 5, left lateral 5), and strain magnitude in the lateral subregions often exceeded that of the midsagittal subregions. The magnitude of longitudinal muscle strain did not vary consistently across the vertebral column (Fig. 5).

### Comparison of predicted and measured muscle strain

**Fig. 6.**
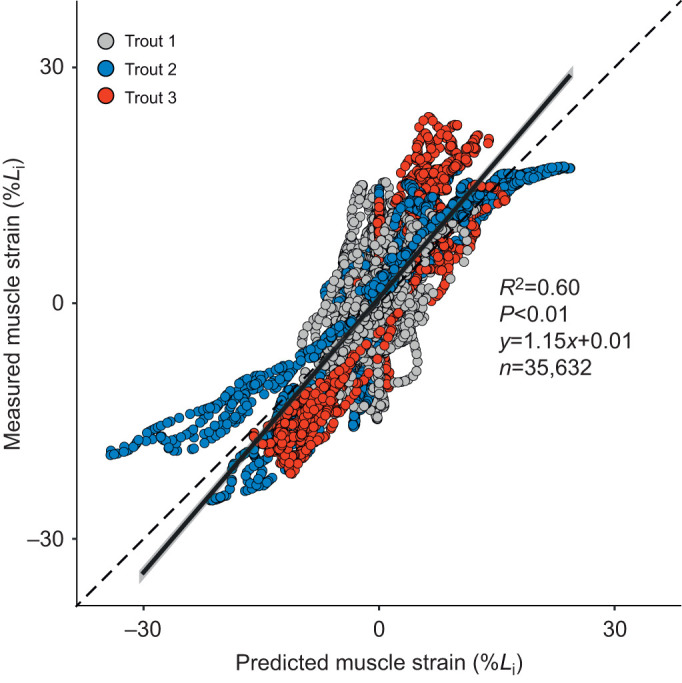
**Relationship between predicted and measured muscle strain confirms beam-like bending.** Linear regression statistics and 95% confidence intervals (grey shading around regression line) for the pooled data (i.e. including all time points, strikes, marker pairs and individuals). The solid line is the best-fit model for our data and the dashed line is the theoretical relationship predicted by beam theory. The number of data points in this plot was reduced by 50% to aid visualization.

We used linear regression to test whether beam theory accurately predicted longitudinal muscle strain, relative to the strains measured with fluoromicrometry (Fig. 6). We found a strong and significant correlation between predicted and measured strain for pooled data across all strikes and subregions (*n*=35,632, *R*^2^=0.60, *P*<0.01; Fig. 6), where the fitted regression model was measured strain=1.15×predicted strain+0.01. If beam theory was perfectly accurate, the slope of this regression would be 1 and the *y*-intercept would be 0. Our regression model fell outside these values for perfect accuracy, based on the 95% confidence intervals of the regression model. However, we performed an equivalence test (bounds: slope±0.2 and *y*-intercept±0.05) and found that our regression model for feeding is statistically equivalent to prior beam theory work in fish swimming (Coughlin et al., 1996; Goldbogen et al., 2005; Katz et al., 1999; Long et al., 2002; Wakeling and Johnston, 1999).

**Fig. 7.**
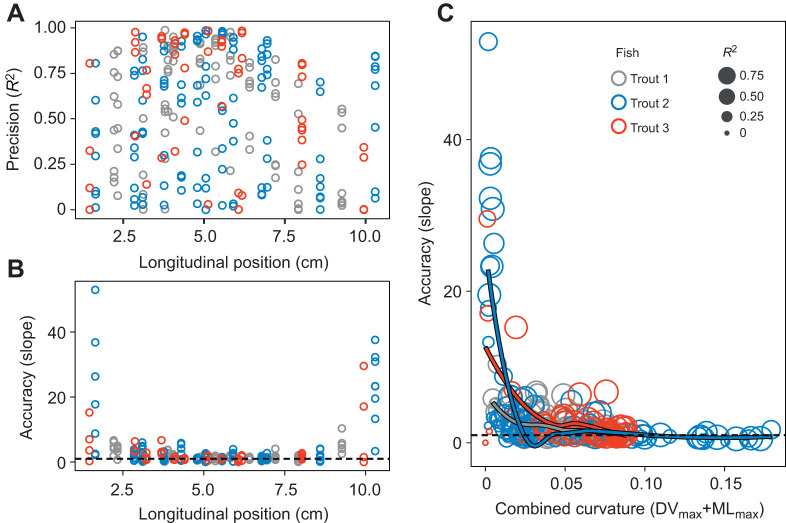
**Accuracy and precision of beam-theory relationship per strike and per subregion as a function of subregion position and curvature.** (A) Precision, i.e. *R*^2^ of linear regression, as a function of longitudinal position (0 cm=craniovertebral joint). (B) Accuracy, i.e. slope of linear regression, as a function of longitudinal position, where perfect accuracy is a slope of 1 (horizontal dashed line). (C) Accuracy as a function of vertebral curvature for a given strike and subregion. Combined curvature is the sum of maximum absolute mediolateral (ML) and dorsoventral (DV) curvature. Each circle's position indicates the individual per-strike region accuracy, and its size indicates precision (*R*^2^). In all panels, individual trout are indicated by different colours, and each data point represents a single strike within a particular muscle subregion (*n*=280 per-strike subregions).

When compared among subregions, the precision and accuracy of our beam theory model varied with a subregion's craniocaudal position and/or magnitude of vertebral curvature (Fig. 7). Model precision (*R*^2^) showed no relationship with the longitudinal position of the subregion (Fig. 7A). However, precision was linked to the magnitude of curvature during a specific strike. For example, excluding data with a combined maximum curvature less than 0.05 cm^−1^ substantially improved model precision: from *R*^2^=0.60 to *R*^2^=0.75. Model accuracy (regression slope) did vary with subregion position: the cranial-most and caudal-most subregions had lower accuracy (slopes of up to 30–45) than all the rest (Fig. 7B). Like precision, accuracy tended to be lower in subregions that underwent the least curvature (Fig. 7C). But unlike precision, excluding these low-curvature subregions did not affect model accuracy (*n*=16,862, *y*=1.14*x*+0.01, *R*^2^=0.75, *P*<0.01).

## DISCUSSION

Using the highest-resolution data collected so far on *in vivo* vertebral curvature and muscle deformation in fish, we measured strain and curvature in both sagittal (dorsoventral) and frontal (lateral) planes during feeding. This allowed us to demonstrate a mathematical relationship between skeletal motion and longitudinal muscular deformation –beam theory – that could describe even complex 3D motions. Beam theory predicted epaxial muscle strain accurately and precisely for feeding strikes, demonstrating that a dorsoventral strain gradient forms as the vertebral column flexes dorsally. This occurs simultaneously with lateral vertebral flexion – well-known to form a mediolateral strain gradient (Long et al., 2002) – providing further evidence that biplanar body flexion imposes orthogonal strain gradients on fish axial muscles (Jimenez et al., 2021).

### Biomechanical implications of beam-like bending

The match between beam theory's predictions and our muscle strain measurements demonstrates a linear, dorsoventral strain gradient in the epaxial muscles during feeding in trout. Dorsoventral strain gradients were previously observed during feeding in bluegill sunfish, although it could not be determined whether these were linear (Jimenez et al., 2021). In trout, the dorsoventral strain gradient extends over a large region of the vertebral column: at least 30% (20 vertebrae). Our results also confirmed mediolateral strain gradients in this region, as previously shown during swimming in rainbow trout (Goldbogen et al., 2005). These gradients may extend further into the cranial-most and caudal-most regions, but beam theory's predictions were less accurate in these regions (Fig. 7B). Either these regions do not experience beam-like bending and a dorsoventral strain gradient, or the low-magnitude curvature in these regions resulted in lower-accuracy measurements (Figs 4 and 7). The former seems likely for the cranial-most epaxials, which extend over the dorsal surface of the neurocranium (rather than vertebrae). Higher-resolution measurements of muscle deformation in these regions are needed to resolve this question.

Rainbow trout experience two orthogonal gradients of longitudinal strain – dorsoventral and mediolateral – in their epaxial muscles during swimming and feeding. Orthogonal strain gradients were previously found in bluegill sunfish (Jimenez et al., 2021), a distantly related species with different body shape and kinematics. Thus, orthogonal strain gradients are not limited to a single lineage or morphotype, and the preconditions may be present in many non-tetrapod fishes that use cranial elevation. Mediolateral strain gradients are widely observed in swimming fish (reviewed in Long et al., 2002). And a diverse set of fishes use longitudinal epaxial shortening and likely vertebral curvature during feeding (Jimenez et al., 2018; Camp, 2021; Camp and Brainerd, 2022), as in trout and sunfish, suggesting dorsoventral strain gradients are also present in many fishes.

Orthogonal strain gradients raise new questions about the relationship between body shape and axial muscle power output. Fish with dorsoventrally ‘taller’ and mediolaterally ‘wider’ bodies have a greater mass of axial muscle to generate more power but will experience larger strain gradients because the superficial muscle regions are farther from the neutral axis. These larger strain gradients are expected to decrease muscle power output in swimming and/or feeding (Jimenez et al., 2021), unless they can be homogenized at the fibre level. Therefore, body shape may impose performance trade-offs of profound ecological relevance.

Beam-like bending in fish during swimming – and now feeding (this study; Jimenez et al., 2021) – is a puzzling phenomenon from a materials perspective. Beam theory assumes a simple and homogeneous material, but fish bodies are complex and heterogeneous (Katz et al., 1999; Long et al., 2002; Jimenez et al., 2023). The body contains a wide range of materials including skin, bones, joints, the notochord, connective tissues, muscle fibres, nerves and blood vessels. These materials are also arranged in an intricate and hierarchical architecture (Fig. 1D), with W-shaped muscle segments (myomeres) nesting within each other, each containing a wide array of muscle fibre orientations (Alexander, 1969; Gemballa and Vogel, 2002; Greene and Greene, 1914; van Leeuwen et al., 1990, 2008). We found strong support for beam-like bending in all the myomeric subregions measured in this study, despite complex and variable 3D morphologies. This suggests that the musculature as a whole, just like isolated muscles (Morrow et al., 2010), behaves like a transversely isotropic material where physical behaviour is symmetric about the longitudinal axis. How exactly does this heterogeneous structure bend like a simple beam? In swimming, axial tendons are hypothesized to transmit bending forces throughout the axial muscles to couple muscle deformation and vertebral curvature throughout the cross-section (Long et al., 2002). It remains to be determined whether a similar mechanism explains how the heterogenous fish body bends like a homogeneous beam during feeding.

The existing framework of muscle gearing suggests that the axial muscles cannot homogenize fibre strain – and therefore maximize instantaneous power – along both orthogonal gradients (Jimenez et al., 2021). In a muscle, gearing (i.e. the ratio of longitudinal muscle strain to fibre strain) emerges from 3D muscle fibre orientation, 3D muscle tissue deformation, and magnitude of bending (Azizi and Brainerd, 2007; Azizi et al., 2008; Muller and van Leeuwen, 2006). As fibre angles are ‘fixed’ in an adult animal, gearing can only be altered by varying muscle deformation and/or body flexion. In swimming, the muscle at a given mediolateral position has one gear ratio to bring local fibres closer to the length or velocity for maximizing force or power, respectively (Azizi and Brainerd, 2007; Rome and Sosnicki, 1991). If dorsiflexing during feeding involves similar gear ratios, muscle deformation and bending, it is highly unlikely these gear ratios could homogenize the dorsoventral gradient of strain and velocity in feeding. Thus, either (1) muscle power is maximized for either swimming or suction feeding or (2) muscle power is maximized for both behaviours through some unknown anatomical or physiological mechanism (Jimenez et al., 2021). Therefore, if dorsoventral strain gradients in suction feeding are widespread, a new interpretation of axial muscle architecture and physiology is needed to understand how fish generate powerful swimming and feeding behaviours.

### Comparison with swimming behaviours

The strain–curvature relationship during feeding was strong and well explained by beam theory, but the accuracy and precision values differed from those reported for swimming. Beam theory during swimming was generally tested by comparing red or white muscle strain calculated from midline curvature in high-speed video with strain from sonomicrometry measurements (e.g. Coughlin et al., 1996; Katz et al., 1999). These yielded *R*^2^ values of 0.72–0.98 (Katz et al., 1999; Wakeling and Johnston, 1999) and slopes of 1.046–1.225 (Coughlin et al., 1996; Goldbogen et al., 2005; Long et al., 2002), which represent similar accuracy, but higher precision than the present study.

Here, we present possible reasons why our accuracy and precision – although high – differs slightly from prior beam theory work. First, we measured smaller magnitude body motions. Trout can bend far more while escaping (0.05–0.33 cm^−1^), and likely feeding (Konow and Sanford, 2008), than we observed (0.06–0.15 cm^−1^). Nevertheless, beam-like bending occurs in swimming with low and high degrees of flexion (Goldbogen et al., 2005; Wakeling and Johnston, 1999), so we expect beam-like bending would still occur during the maximal curvatures missing from our feeding dataset. Second, we measured curvature from a 3D reconstruction of the vertebral column with biplanar bending rather than a 2D body outline with only lateral bending. Assuming that total vertebral curvature was the sum of curvature in the projected planes decreased vertebral length by 1–2%, relative to resting length, in the projected planes. A calculation of pure 3D vertebral curvature could be beneficial but, in our context, 2D planes are advantageous for describing motion from an anatomical and functional perspective. This highlights both the great opportunities and conceptual challenges created by the recent boom in 3D motion data. Third, the strain–curvature relationship may be slightly but genuinely different in feeding. Fourth, we used the average marker position at rest to calculate muscle subregion location for Eqn 2, yet markers may shift relative to this as a result of normal muscle bulging. Some or all of these may account for the small differences in how precisely beam theory explains our feeding data, compared with previous swimming studies. However, beam theory still robustly describes the relationship between vertebral flexion and muscle deformation during suction feeding.

### Conclusions

We present the most comprehensive measurements of simultaneous longitudinal muscle strain and vertebral curvature collected so far, which show trout undergo similar beam-like body bending in feeding to that in swimming. Our results suggest that beam theory can describe epaxial deformation for vertebral flexion in any direction, and that the epaxial muscles experience two, orthogonal gradients in longitudinal muscle strain – often simultaneously. Further study is needed to determine how fish may overcome these strain gradients to generate powerful swimming and feeding, and whether the competing muscular demands of lateral and dorsal flexion impact feeding performance and strategies (Jimenez and Brainerd, 2021; Jimenez et al., 2021). Discovering how fish axial muscles power 3D vertebral motion will be key to understanding their complex architecture and their contribution to the diversification and adaptation of non-tetrapod fishes.

## Supplementary Material

10.1242/jexbio.245788_sup1 Supplementary information
