# Supplementary material for: Beam theory predicts muscle deformation and vertebral curvature during feeding in rainbow trout (Oncorhynchus mykiss)
Source: J Exp Biol. 2023 Oct 31;226(20):jeb245788. doi: 10.1242/jeb.245788 (PMC10629686; doi:10.1242/jeb.245788)
Supplement: Supplementary information [file jexbio-226-245788-s1.pdf]

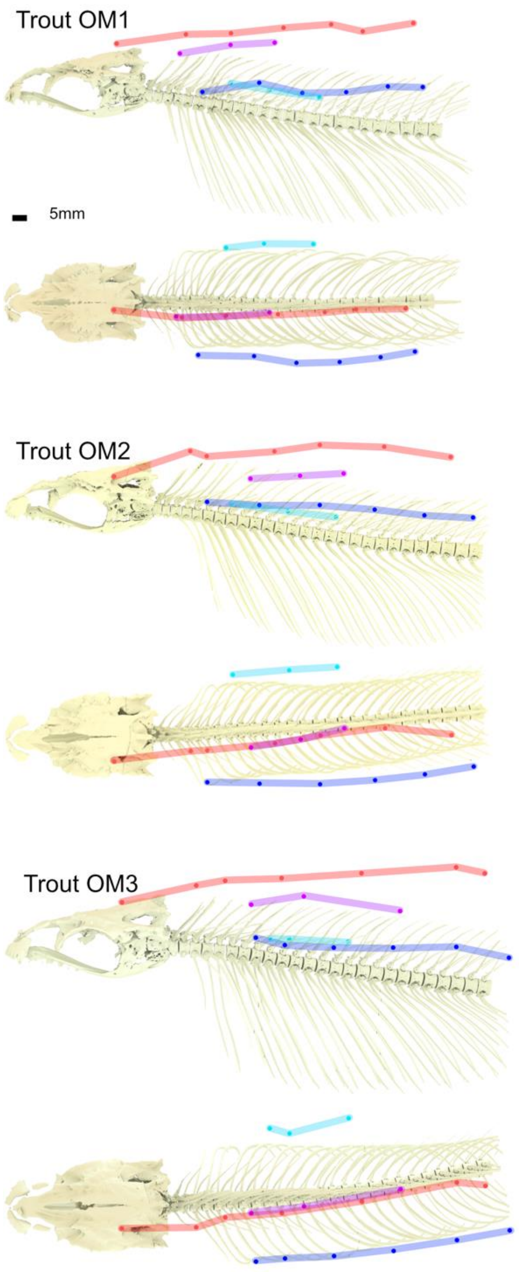

Fig. S1. Muscle marker placement, relative to craniovertebral skeleton, for each individual trout. Images are rendered from orthographic dorsal and lateral camera views. For each individual, markers are shown at the first frame, before the onset of the strike, of the trial with the least lateral and/or dorsal vertebral flexion at this time. This "initial position" was used to calculate the dorsoventral and mediolateral distance of each marker from the vertebral column and the initial length (L_i) of each muscle subregion (see Fig. 3 and Methods for more details).

Journal of Experimental Biology • Supplementary information
